# Non-Alcoholic and Low-Alcohol Beer: Regulatory Complexity, Market Expansion and Technical Bottlenecks in Biological and Physical Dealcoholisation

**DOI:** 10.3390/foods15162822

**Published:** 2026-08-13

**Authors:** Alessia D’Andrea, Francesco Licciardo, Erika Celi, Katya Carbone

**Affiliations:** 1 Centro di Ricerca Olivicoltura, Frutticoltura, Agrumicoltura, Consiglio per la Ricerca in Agricoltura e l’Analisi dell’Economia Agraria, Via di Fioranello 52, 00134 Rome, Italy; 2 Centro di Ricerca Politiche e Bio-Economia, Consiglio per la Ricerca in Agricoltura e l’Analisi dell’Economia Agraria, Via Barberini 36, 00187 Rome, Italy; francesco.licciardo@crea.gov.it

**Keywords:** no-alcohol and low-alcohol beer, dealcoholisation, NABLAB, beverages, beer, No-Lo

## Abstract

The rising health consciousness among Millennials and Generation Z is boosting the demand for non-alcoholic and low-alcohol beers (NABLABs), which offer the sensory appeal of beer with fewer ethanol drawbacks. This study aims to provide a comprehensive overview of this sector, covering market trends, regulatory complexities, and the technical aspects of biological and physical dealcoholisation methods, and offering insights into production costs and environmental impact. The global market for NABLABs is experiencing rapid growth, with a projected compound annual growth rate of approximately 7% (2024–2028) and an estimated market value approaching USD 44 billion by 2034. Market penetration remains geographically heterogeneous, and there is a lack of regulatory harmonisation, particularly within the European Union, where national thresholds for ethanol content classification diverge significantly. From a process engineering perspective, NABLAB production can be achieved using two main technological approaches: (i) biological ethanol limitation strategies, encompassing controlled mashing, arrested fermentation, cold-contact fermentation and the use of maltose-negative or genetically modified yeast strains, and (ii) post-fermentation dealcoholisation, employing thermal separation technologies, such as vacuum distillation, thin-film evaporation and spinning cone columns, or membrane-based processes such as reverse osmosis, nanofiltration, pervaporation, osmotic distillation and dialysis, alongside emerging supercritical CO_2_ extraction. A critical technological trade-off persists between the efficiency with which ethanol is removed and the retention of volatile flavour-active compounds, including esters and higher alcohols. It is evident that biological processes are associated with the accumulation of worty aldehydes and the attenuation of antimicrobial barriers, resulting in increased risks to microbiological safety. Empirical evidence has been documented showing that *E. coli* O157:H7, *Salmonella enterica*, and *Listeria monocytogenes* have been found to survive for extended periods in craft NABs exhibiting pH > 4.20 and an alcohol-by-volume (ABV) < 0.5%. This emphasises the importance of validated thermal or sterile filtration treatments, particularly in the craft beer sector.

## 1. Introduction

There is increasing interest among younger generations in healthy lifestyles and a growing demand for low-alcohol and non-alcoholic (NoLo) alternatives to traditional alcoholic beverages, such as wine, cider, and beer [1,2], with the latter being one of the most widely consumed beverages worldwide [2,3].

China, the United States, and Brazil account for approximately 40% of the global beer production. China remains the world’s leading producer, maintaining an annual output of approximately 36 billion litres (360 million hectoliters), which represents roughly 20% of the global market, with most of its output being consumed domestically [4].

Despite the rapid market growth, the regulatory landscape for NABLAB remains complex and fragmented. Currently, no universally accepted standard governs its definition, labelling, or marketing across jurisdictions. This regulatory fragmentation poses significant challenges for manufacturers operating in multiple markets, as it requires a comprehensive understanding of the specific requirements applicable in each region.

For a beverage to be classified as non-alcoholic (NA) or low-alcoholic (LA), it must have an alcohol-by-volume (ABV) content within a certain range, as determined by competent authorities in each country. Currently, most laws worldwide define NABs as containing ≤0.5% ABV and LABs as containing ≤ 1.2% ABV [5,6]. In some countries, such as Brazil, the ABV of LABs can reach 2% [7].

Countries in the European Union (EU) generally adhere to the worldwide standard, although with some exceptions (e.g., Spain, Sweden, Finland, and Austria have higher than average alcohol thresholds). Italy, in particular, classifies any beer with an alcohol content lower than 1.2% ABV as NA [8]. Nevertheless, a legal definition for NoLo beverages is currently absent in the European Union.

However, the complexity goes well beyond the ABV thresholds used to define the product category and encompasses at least four largely independent regulatory dimensions: labelling, biotechnology/GMO approval, marketing and advertising, and tax/excise classification. Each of these areas may apply different alcohol content thresholds and levels of harmonisation, thereby increasing the compliance burden on producers. Beyond the ABV threshold itself, labelling regimes determine what claims a NABLAB may legally make and what information must accompany them. These requirements diverge considerably across jurisdictions. In the EU, Regulation (EU) No 1169/2011, on the provision of food information to consumers, governs mandatory information and ingredient and allergen declarations [9]. It also sets out the conditions under which voluntary claims, such as ‘alcohol-free’ or ‘0.0%’, may be used. However, the regulation itself does not harmonise the ABV threshold at which such claims become permissible. Instead, it leaves this to national implementation. This results in the same fragmentation at the labelling level that was previously described for product classification. Terminology is another source of confusion: some markets distinguish between “alcohol-free” (which implies the lowest possible residual ethanol content, typically <0.05% ABV) and “non-alcoholic” or “low-alcohol” (which allow for a residual ethanol content up to the thresholds of 0.5% or 1.2% mentioned above), while others use these terms interchangeably, creating potential confusion among consumers when products are marketed across borders. In several jurisdictions, including the United States, products with an alcohol content below 0.5% ABV are also subject to health warnings regarding pregnancy, regardless of whether they are labelled as “non-alcoholic,” reflecting the non-zero residual ethanol content typical of most NABLAB products produced through both biological and physical dealcoholisation processes, as discussed in the following sections. Taken together, these labelling differences mean that a single formulation may require different front-of-package claims, different mandatory warnings, and different ingredient information depending solely on the market in which it is sold, regardless of any changes to the product or the underlying production process.

Marketing restrictions introduce an additional layer of complexity that often does not align with the ABV thresholds used for product classification and taxation. The World Health Organization’s Global Action Plan on Alcohol 2022–2030 explicitly recommends that Member States consider extending marketing and advertising restrictions to NoLo products that are branded, packaged or promoted in a way that closely resembles alcoholic beverages. This is because such products could normalise alcohol brands, particularly in the eyes of minors, even when the product itself falls below the legal alcohol content threshold [10]. This creates a regulatory paradox in several jurisdictions: a NABLAB may fully meet the compositional requirements to be labelled as ‘non-alcoholic’, yet still be subject to the advertising restrictions applicable to conventional alcoholic beverages simply because of its brand lineage. This means that compliance with the alcohol content requirement alone does not guarantee freedom from restrictions.

Fiscal treatment constitutes another regulatory dimension that is largely independent and has direct commercial consequences for retail pricing and competitiveness. Within the EU, Council Directive 92/83/EEC, as amended by Council Directive (EU) 2020/1151, permits Member States to apply reduced or zero excise duty rates to beer with an ABV of no more than 0.5%. However, national implementation is subject to considerable discretion, meaning that the fiscal threshold does not always coincide with the labelling or marketing thresholds discussed above [11]. The United Kingdom’s 2023 alcohol duty reform shifted towards a strength-based duty structure. It set the threshold for products entirely outside the scope of alcohol duty at 1.2% ABV rather than the more commonly used 0.5% threshold for “non-alcoholic” classification elsewhere. This means that products with an ABV between 0.5% and 1.2% are treated as duty-free in the UK, although they may still be labelled as “low-alcohol” rather than “non-alcoholic” in other markets [12]. In the United States, products with an ABV below 0.5% fall outside the definition of a “malt beverage” that is subject to federal alcohol excise tax, as administered by the Alcohol and Tobacco Tax and Trade Bureau (TTB). This shifts primary regulatory oversight to the Food and Drug Administration (FDA), altering both the applicable labelling regime and the retail channels through which such products may be sold (e.g., grocery and convenience stores without an alcohol licence) [13]. Outside Europe and North America, two of the largest NABLAB markets demonstrate the significant divergence of classification thresholds. In Japan, the Liquor Tax Act (Act No. 6 of 1953), administered by the National Tax Agency, defines an alcoholic beverage as any beverage with an alcohol content of 1% ABV or above [14]. Beverage alcohol content falling below this threshold is regulated as soft drinks under the Food Sanitation Act. Consequently, they are exempt from liquor tax and do not require a liquor retail licence. This is one of the factors contributing to the convergence of the Japanese “beer-taste beverage” segment, which has largely adopted 0.00% ABV formulations instead of the 0.5% ceiling observed in other markets. In the Australian context, on the other hand, the Australia New Zealand Food Standards Code stipulates that any beverage with an alcohol content of 0.5% ABV or more is required to explicitly declare its alcohol content and to include a standard-drinks statement [15]. For beverages with an ABV of 1.15% or less, the declaration must be expressed in words as “contains not more than X% alcohol by volume”. For beverages with an ABV above 1.15%, the declaration must be expressed as a percentage of ABV or in mL per 100 mL. The same 1.15% threshold governs the mandatory pregnancy warning label, the ability to make nutrition content claims, and the prohibition on representing a product as a “low alcohol beverage”. In addition, the Schedule to the Excise Tariff Act 1921 stipulates that excise duty is applied to beer only when the alcohol content exceeds 1.15% ABV [16]. Consequently, beers with a strength below or equal to this limit are exempt from duty.

When considered alongside the European Union (with national derogations ranging from 0.5% to 1.2%), the United Kingdom (with a duty threshold of 1.2%) and the United States (with a TTB/FDA boundary of 0.5%), it is clear that there is no single global cut-off point. At least four distinct regulatory thresholds (0.5%, 1.0%, 1.15% and 1.2% ABV) are currently in use simultaneously, and different thresholds may apply within a single jurisdiction for classification, labelling, marketing and excise purposes. For a producer marketing one formulation internationally, this determines not only the label, but also the degree of dealcoholisation that must be achieved and, therefore, the process technology that must be selected.

In addition to market and regulatory considerations, therefore, producing NABLABs presents significant scientific and technological challenges. This is because ethanol is not just a diluent but also plays a key role in determining the body, mouthfeel, aroma balance, foam stability and microbiological robustness of beer [17]. There are currently two main technological routes employed to reduce or remove ethanol from beer: (i) the physical de-alcoholisation of conventionally fermented beer, using either thermal methods, such as vacuum distillation and spinning-cone evaporation, or membrane-based methods, such as reverse osmosis, dialysis or osmotic distillation [17]; and (ii) biological approaches that limit ethanol formation during fermentation itself. These include arrested or restricted fermentation, cold-contact processes, limited-mashing processes, and the use of non-conventional, low-fermenting yeasts such as *Saccharomycodes ludwigii*, *Zygosaccharomyces rouxii* and selected *Torulaspora* and *Pichia* species, or engineered maltose-negative *Saccharomyces* strains [18,19]. Each strategy has its own set of trade-offs. Thermal dealcoholisation is energy-intensive and tends to strip away volatile aroma compounds, resulting in undesirable cooked or caramelised notes. Membrane processes are milder but costly and difficult to scale up [17]. Biological methods often produce a pronounced ‘worty’, cereal-like off-flavour associated with the incomplete fermentation of wort aldehydes (e.g., 3-methylbutanal) and insufficient esterification. There is also reduced microbial stability due to the reduced antimicrobial effect of ethanol [20]. Recent literature has identified several key innovations that are needed, such as optimising and integrating hybrid processes that combine controlled fermentation with mild membrane or thermal dealcoholisation; developing natural flavour-masking and aroma-recovery strategies [21]; and implementing non-thermal stabilisation technologies (e.g., high-pressure processing and cold sterile filtration) to safeguard microbiological quality without further compromising sensory attributes [20]. Moreover, key innovations also include screening and metabolic or genetic engineering of novel non-*Saccharomyces* and hybrid yeast strains with improved flavour profiles. However, this latest technological approach brings to light another relatively unexplored aspect of regulatory complexity: the legal status of yeast strains used in biological dealcoholisation (Section 4.1.4). Maltose-negative and low-fermentation strains obtained through classical mutagenesis or adaptive evolution in a laboratory setting, including spontaneous mutants of *Saccharomyces pastorianus* and *non-Saccharomyces* species found in nature [22,23,24], are generally not subject to EU GMO regulations, as organisms obtained through mutagenesis are explicitly exempt from the authorisation requirements set out in Directive 2001/18/EC on the deliberate release of genetically modified organisms, provided the technique has a long safety track record [25]. In contrast, strains obtained through recombinant DNA technology or transgenic modification must undergo a full risk assessment on a case-by-case basis. They are also subject to authorisation, traceability and labelling requirements under Directive 2001/18/EC and Regulation (EC) No 1829/2003 on genetically modified food and feed. This is a substantially more demanding and costly regulatory pathway, which has limited the commercial use of transgenic yeast strains in EU beer production to date [25,26]. However, this binary, process-based approach is currently under revision. The European Commission’s 2023 proposal for a regulation on plants (and, by extension, microorganisms) obtained by certain new genomic techniques (NGTs) would exempt a defined category of gene-edited organisms, those bearing genetic changes that could occur naturally or through conventional breeding, from the full GMO authorisation pathway. This could ease regulatory access for precision-edited, non-transgenic yeast strains with improved maltose-negative or flavour-enhancing traits [27]. This contrasts with the product-based regulatory philosophy applied in the United States and several other jurisdictions, where the characteristics of the final organism or product generally determine the regulation, rather than the technique used to obtain it [28]. Consequently, a non-transgenic, genome-edited yeast strain may not automatically require GMO-specific authorisation or labelling. This transatlantic divergence in biotechnology governance is a significant and largely unaddressed source of regulatory complexity for brewers seeking to deploy the next generation of engineered low-fermenting yeast strains.

Therefore, addressing all these bottlenecks is essential if we are to sustain the sensory quality, safety and continued market growth of NABLABs.

In this context, this review aims to carry out a critical analysis of current production strategies for NoLo beers, including the technological limitations associated with these strategies, as well as emerging opportunities for research and innovation in the sector. Furthermore, in order to provide as comprehensive a picture of the sector as possible, it also aims to offer an overview of trends in the NABLAB market, providing insights into production costs and environmental impact.

## 2. Methodology

The methodological approach was based on a comprehensive review of the scientific literature and technical and regulatory documentation available online. Three source types were used, which are not treated as equivalent. Peer-reviewed literature constitutes the primary evidence base, carrying all technical, microbiological, and process-related claims. It was identified in Scopus on 14 April 2025 using the following query: TITLE-ABS-KEY ((“low-alcohol” OR “low alcohol” OR “alcohol-free” OR “non-alcoholic” OR “low-alcoholic” OR “NoLo” OR “no-alcohol” OR “NABLAB”) AND beer). AND PUBYEAR > 2018 AND LIMIT-TO (LANGUAGE, “English”) AND LIMIT-TO(EXACTKEYWORD,”Beer”), which returned 264 records. The 2019–2025 timeframe was selected to encompass the period during which the category experienced commercial growth. Seminal earlier works were identified through backward citation tracking, and the search was updated using forward citation tracking up to June 2026 to incorporate studies published during the revision process. Primary regulatory sources, such as Directives, Regulations, national statutes, and guidance from competent authorities (e.g., the European Commission, TTB, FDA, the Japanese National Tax Agency, etc.), were consulted in their official, consolidated versions and are cited directly. As they are legal instruments rather than bibliographic records, they were identified through legal citation chains rather than keyword searches. Finally, commercial market intelligence reports (e.g., from IWSR, GlobalData and Mordor Intelligence) were used exclusively to provide up-to-date estimates of market size, growth and penetration. This is because comparable, regularly updated global market data are generally unavailable in peer-reviewed literature. Due to the proprietary nature of their methodologies, the market figures derived from these reports are presented as indicative estimates and should be interpreted alongside the available scientific literature.

## 3. Market Context and Growth Drivers

The growth of the NABLAB market cannot be attributed solely to increasing product availability. It also reflects broader changes in drinking behaviour, including growing interest in health-conscious lifestyles and more moderate patterns of alcohol consumption [29,30]. These trends have been particularly evident among younger consumers, whose purchasing decisions are increasingly influenced by health-related considerations [31]. At the same time, advances in brewing and dealcoholisation technologies have progressively narrowed the sensory gap between conventional and alcohol-free beers, facilitating wider market acceptance and encouraging breweries to expand their NABLAB portfolios [32,33].

Sales volume data confirm this upward trajectory: global consumption of NAB surpassed 2.5 billion litres in 2024 and is estimated to reach approximately 2.7 billion litres by the end of 2025, representing an overall volume increase of more than 40% compared to 2018 levels [34]. This surge in consumption is not limited to a single region; rather, the ten largest markets, including Australia, Brazil, Canada, France, Germany, Japan, South Africa, Spain, the United Kingdom and the United States, collectively added over 61 million new NAB consumers and 38 million new LAB consumers between 2022 and 2024 [35]. Economically, the growing market share of NABLABs is reshaping the competitive landscape, with projections suggesting that NA products will account for over 3% of the total beverage alcohol market volume by 2028 [35].

The market evidence is consistent with the main drivers identified in the literature, namely increasing health consciousness and moderation-oriented lifestyles [29,30,36], continued product innovation aimed at improving the sensory quality of NABLAB products [32,33,37,38], broader product availability through both traditional retail and e-commerce channels [34], and strategic responses adopted by breweries to address evolving regulatory and sustainability requirements [39]. In mature beer markets, where overall beer consumption has remained relatively stable, these developments have reinforced the strategic importance of NABLAB products within brewers’ portfolios [35].

Taken together, these factors provide a useful framework for interpreting the market trends discussed below and the varying rates of NABLAB adoption across countries. They also suggest that NABLABs should no longer be regarded as a niche segment but rather as an established component of the evolving global beer market.

However, as shown in Table 1, the growth trajectories and market penetration of NABLABs significantly vary across countries. Germany exhibits a highly mature market, where the share of NAB in total beer sales exceeds 14% and is expected to maintain this dominance through 2029. Other European nations, including Spain, the Czech Republic and France, along with Japan, constitute a robust mid-tier market, with NABLAB’s market shares projected to grow steadily to reach between 6% and 9% by 2029. Conversely, markets such as the United Kingdom, the United States, and Brazil show much slower adoption rates, with market shares lingering at or below the 2% threshold [40]. Furthermore, although China remains the world’s largest beer market, NAB consumption there remains relatively limited. The Chinese market does not yet appear to be significantly influenced by the broader global shift towards NABLAB consumption, a phenomenon largely attributed to deep-rooted cultural factors wherein traditional alcohol consumption is intricately tied to social bonding and business etiquette [41,42].

In addition to the rising consumer demand for NABs, it is essential to consider the medium-term impact of government regulations on advertising restrictions, labelling requirements, and alcohol content limits on the beer market over time (Table 2). According to recent scenario analyses [39], in fact, potential regulatory constraints could lead to a reduction of up to 0.80% in the CAGR of the traditional beer market over the medium term.

However, while stringent policies may constrain the production volumes of full-strength beer, the concurrent shift in consumer preferences toward health- and wellness-oriented products is driving profound market segmentation. This dual pressure catalyses innovative pathways, including the formulation of beverages with novel ingredients such as functional herbs, spices, and fruits. Ultimately, the increasing demand for NABLAB alternatives represents a vital growth trajectory for the brewing industry, offering critical opportunities for product differentiation and portfolio expansion.

## 4. NABLAB Production Methods: Technical Analysis and Bottlenecks

**Figure 1:**
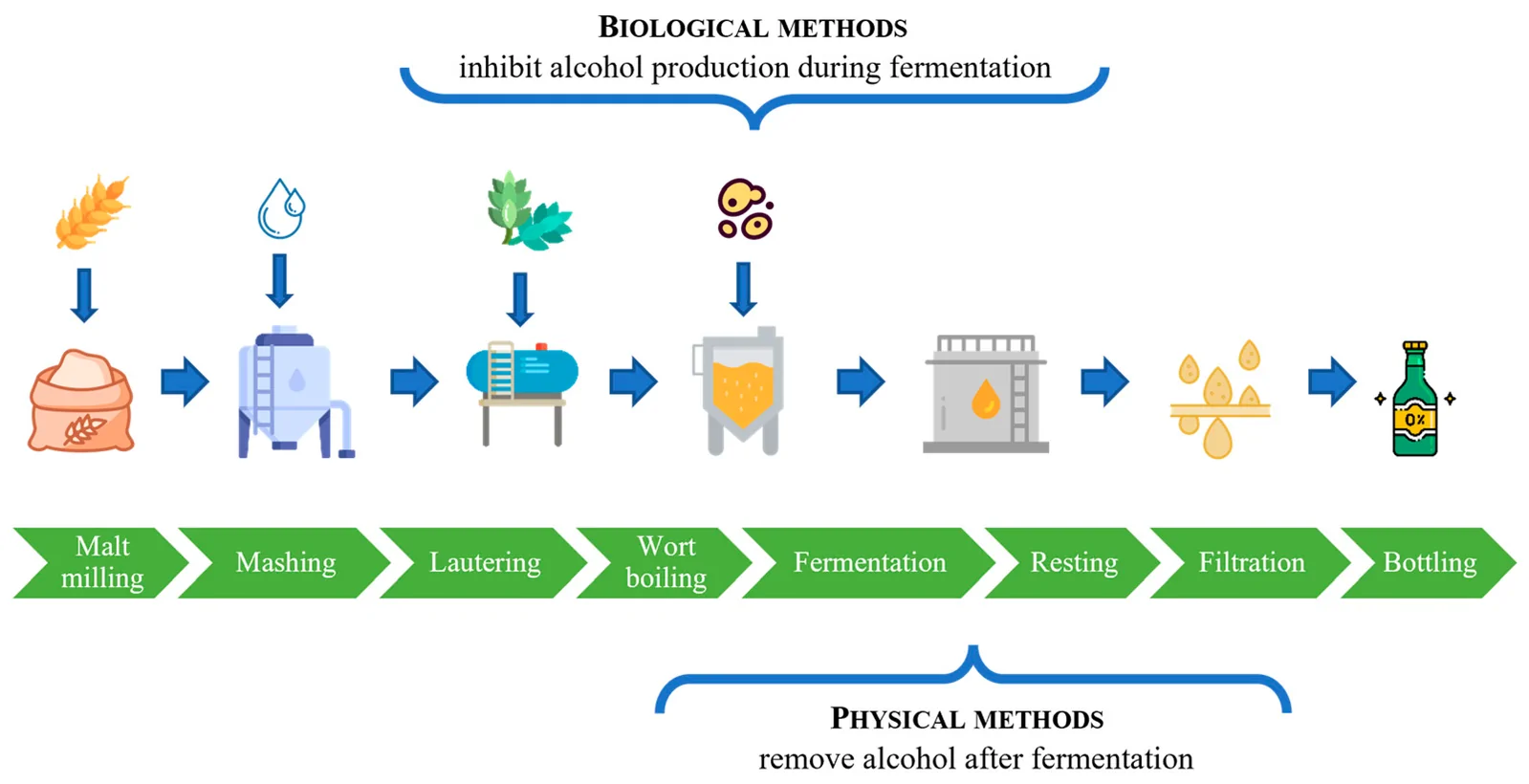
Scheme for traditional brewing and NABLABs production techniques. Image designed using resources from Flaticon.com distributed under the Free Licence (individual icons created by Magnific, Flat Icons, Icongeek26, Sudowoodo, Slamlabs, Vectoricons, Umeicon, and bsd).

Producing high-quality NABLAB is a delicate process that requires precise management of alcohol removal and preservation of sensory complexity. Thus, several methods have been developed to remove alcohol from beer during the traditional brewing process (Figure 1) [2,43].

**Figure 2:**
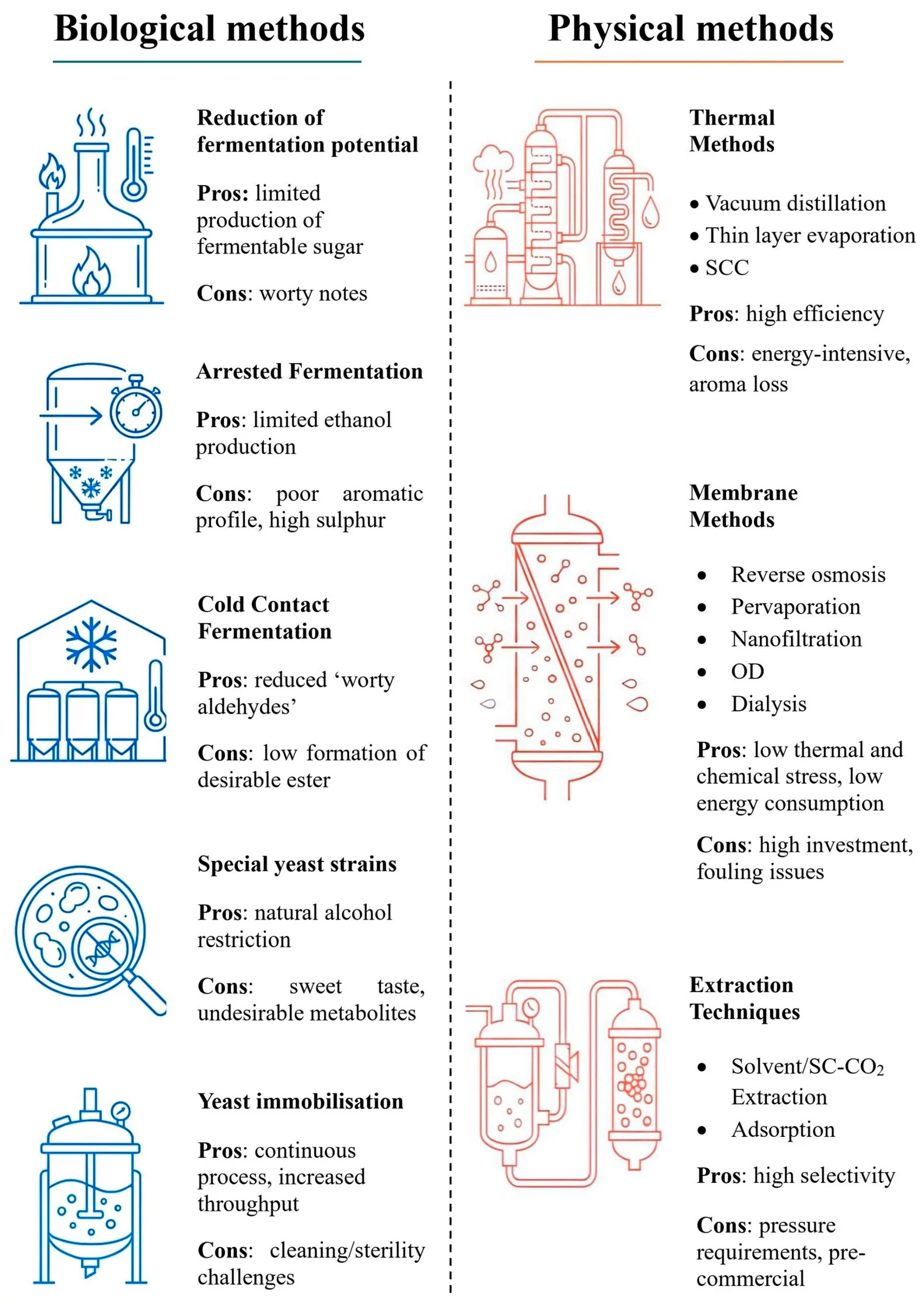
Overview of the main biological and physical methods utilised to obtain non-alcoholic and low-alcoholic beer. For each approach, the key technological advantages (Pros) and limitations (Cons) are highlighted.

They can be divided into two main categories: biological methods (designed to prevent ethanol formation), and physical methods (intended to remove ethanol post-fermentation) [2,33,43,44], each with advantages and disadvantages in terms of alcohol removal efficiency, flavour preservation, energy consumption, and commercial feasibility (Figure 2).

### 4.1. Biological Methods

Biological (or biotechnological) dealcoholisation focuses on preventing alcohol formation during fermentation, rather than removing it afterwards. These methods are widely used to produce craft non-alcoholic beers because they better preserve the flavour profile and complexity than physical techniques do. Furthermore, no alterations to the production facility are required. The main biological methods of dealcoholisation are outlined below.

#### 4.1.1. Reduction in Fermentation Potential (Mashing)

**Figure 3:**
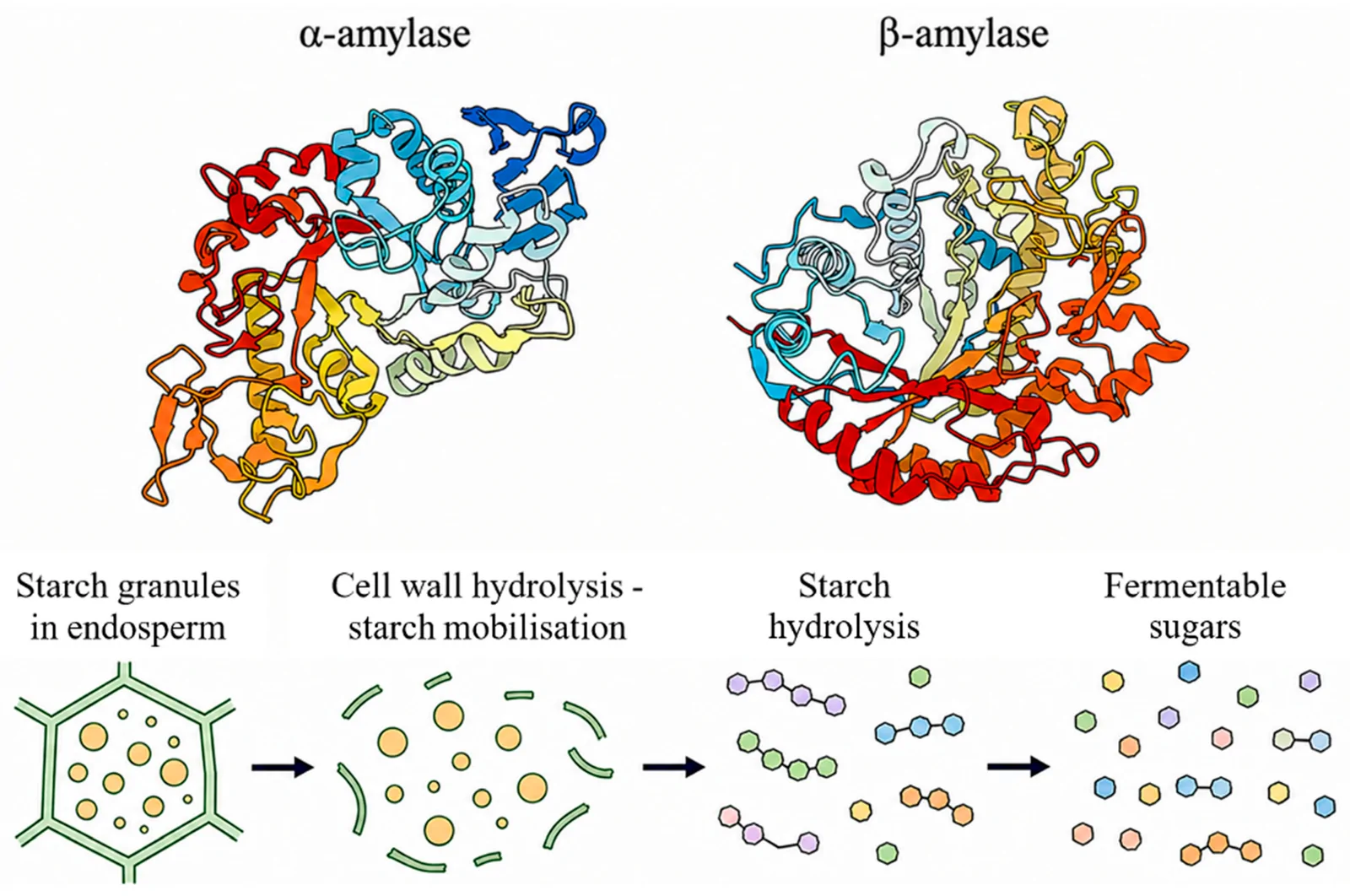
Schematic representation of the enzymatic activity of α-amylase and β-amylase, the main hydrolases involved in the breakdown of starch into fermentable components. The upper panel displays the three-dimensional structures of barley α-amylase (PDB ID 1AMY) and barley β-amylase (PDB ID 2XFR), rendered with the Mol* viewer based on coordinates deposited in the RCSB Protein Data Bank (RCSB PDB). The structures correspond to those reported by Kadziola et al. (1994) [45] and Rejzek et al. (2011) [46], respectively. The lower panel illustrates the main steps of starch degradation process.

This technique is applied prior to fermentation, acting directly on the wort production by limiting the breakdown of starch into fermentable sugars (low-molecular-weight molecules such as maltose and glucose) through inactivation of the amylolytic enzymes (α- and β- amylases) present in barley malt (Figure 3). These enzymes are essential for converting starch into fermentable sugars and dextrins necessary for beer production. It is important to note that β-amylase (optimally active at 55–65 °C and fully inactivated at ~72 °C), which decomposes starch at lower temperatures, must be managed alongside α-amylase, which has a higher optimal temperature range (~70–75 °C, inactivated above 80 °C).

β-amylase is the key enzyme in the production of maltose, the main fermentable sugar in wort. However, it is highly thermolabile, making it susceptible to inactivation at temperatures as low as 62 °C. In contrast, α-amylase is more thermostable, generally maintaining its activity until temperatures reach approximately 72 °C, and it breaks down starch into a mixture of sugars and larger dextrins [47]. The thermal stability of starch-hydrolysing enzymes, particularly β-amylase, is crucial to the yield of fermentable sugars during mashing and, consequently, in determining the final carbohydrate composition of the resulting beer. Inactivating these enzymes, for instance, by mashing at temperatures above 75 °C, restricts the production of fermentable maltose and glucose. Other strategies include the use of unmalted raw materials or the extraction of spent grains with cold water [2], yielding a sweeter beer with a pronounced “worty” flavour [48].

#### 4.1.2. Arrested Fermentation

Arrested fermentation (AF) is a biological method that involves deliberately halting the yeast’s metabolism before a significant amount of alcohol is produced. This can be achieved through one of the following approaches: rapid wort cooling, yeast removal before complete attenuation, or direct fermentation at low temperatures. However, because fermentation is curtailed before complete attenuation, the wort is only partially converted, and the resulting aromatic profile is typically poor, often characterised by residual worty notes. This shortcoming can be partially addressed by incorporating flavouring agents at the end of the brewing process [48]. A further analytical feature of AF is its characteristically elevated sulphur content. Sulphur-containing compounds that would normally volatilise during prolonged fermentation are retained, making dimethyl sulphide a useful analytical marker for this production method [2,49]. It is currently the most widely used method for producing craft beers with low or no alcohol content.

#### 4.1.3. Cold Contact Fermentation

Cold contact fermentation (CCF), also referred to as the cold contact process (CCP), is a controlled, continuous process in which yeast remains in contact with the wort throughout, but its metabolic activity is kinetically suppressed from the outset by maintaining temperatures at or near 0 °C (typically 0–8 °C) for contact times of 24 to 100 h [43]. The key difference between AF and CCF lies in the design of the process: the former terminates an ongoing fermentation, whilst CCF deliberately prevents full fermentative activity from developing in the first place. A key advantage of CCF over AF is that, despite limiting ethanol synthesis, low-temperature conditions do not equivalently suppress all yeast reductive enzyme activities. In particular, yeast aldehyde reductases remain partially active at near-zero temperatures, enabling a degree of reduction in wort-derived Strecker aldehydes, such as methional, 2-methylbutanal, and 3-methylbutanal, which are the main contributors to the raw, grainy, and “worty” off-flavour characteristics of NABs [33,50]. The capacity for aldehyde reduction is largely absent in AF, where a short fermentation window results in the presence of these compounds at concentrations above their sensory thresholds (on average 0.5 µg/L for methional, 23.4 µg/L for 2-methylbutanal, and 0.61 µg/L for 3-methylbutanal) [33,50]. Therefore, CCF produces beers with fewer off-notes than AF, although the formation of desirable esters and higher alcohols, such as isoamyl acetate, ethyl acetate and 2-phenylethanol, is severely limited under cold conditions [33]. The use of CO_2_ overpressure during CCF also helps suppress the volatilisation of sulphur-containing compounds that would otherwise be stripped from the wort during standard fermentation, thereby retaining these compounds in the final beer. Consequently, CCF-produced NABs have elevated sulphur content and characteristically higher pH than conventionally fermented beers, along with elevated levels of Strecker aldehydes, particularly methional [43]. These features represent the primary flavour liabilities of CCF and distinguish its sensory profile from that of both AF and post-fermentation dealcoholised products.

#### 4.1.4. Special Yeast Strains

Special yeast strains used in NABLAB production are selected based on their limited or absent capacity to ferment the principal sugars present in wort, primarily maltose, which typically constitutes 50–60% of wort fermentable carbohydrates. This approach is fundamentally different from AF or CCF in that the restriction of ethanol production is an intrinsic biological property of the yeast itself rather than being imposed by external process conditions. The strains employed can be divided into two conceptually distinct groups. The first comprises genuinely non-*Saccharomyces* species that are naturally maltose-negative, that is, they lack the genetic machinery to transport and metabolise maltose, including *Saccharomycodes ludwigii*, *Zygosaccharomyces rouxii*, *Mrakia gelida*, *Cyberlindnera* spp, *Pichia kluyveri* and *Pichia farinosa* [22,23,24,43]. Because these species cannot utilise the dominant wort sugar, ethanol production is inherently constrained, and only simple sugars, such as glucose and fructose, are consumed during fermentation. The second group consists of spontaneous mutants derived from the conventional lager-brewing yeast *Saccharomyces pastorianus*. Unlike the non-*Saccharomyces* species listed above, which are wild or environmental yeasts exploited for their natural sugar utilisation limitations, spontaneous mutants of *S. pastorianus* are strains that have acquired, through random, unguided genetic mutation (i.e., without deliberate genetic engineering), a reduced capacity to ferment maltose or other wort sugars. *S. pastorianus* is an interspecific allotetraploid hybrid of *S. cerevisiae* and the cold-tolerant *S. eubayanus*, which arose naturally around the 17th century and is responsible for conventional lager beer production [22]. Spontaneous mutants selected from this species retain their established technological properties, such as cold tolerance and flavour-forming capacity, while exhibiting attenuated fermentative vigour, making them of industrial interest for NABLAB production [23].

Irrespective of the strain category, the limited sugar consumption that characterises all these approaches often results in a high residual sugar content in the final product, leading to an excessively sweet taste [49]. Furthermore, the use of genetically modified *Saccharomyces* strains, as opposed to the spontaneous mutants or naturally maltose-negative species described above, carries the additional risk of overproducing undesirable metabolites such as acetoin, acetaldehyde, and diacetyl, imparting a sherry-like character rather than a beer-like flavour profile [43].

#### 4.1.5. Yeast Immobilisation

Yeast immobilisation involves controlling the contact time between yeast and wort by passing the wort slowly and continuously over a fixed bed of yeast cells attached to, or entrapped within, a support material. The advantages of yeast immobilisation include process continuity, increased throughput, and greater biomass economy. Yeast immobilisation can be combined with CCF to further restrict ethanol formation while maintaining aldehyde reduction activity. The four principal immobilisation strategies are the following: (i) adsorption onto a solid surface, such as porous ceramic, spent grains, or corncob carriers; (ii) physical entrapment within a porous polymer matrix, such as calcium alginate or alginate–chitosan microcapsules; (iii) self-aggregation through flocculation in gas-lift bioreactors; and (iv) containment behind a semi-permeable membrane barrier [17,49]. Each approach presents distinct trade-offs in terms of mass transfer efficiency, yeast physiology, flavour compound formation, and operational scalability. Although immobilisation systems are more time-efficient than batch methods and allow for easy yeast recovery and reuse, they are difficult to optimise, necessitate specialised continuous bioreactor infrastructure, and are more challenging to clean and validate for sterility than traditional batch vessels.

#### 4.1.6. Biological Methods: Limitations

Collectively, biological pre-processing methods share a common set of limitations that have significant implications for product quality and food safety. From a quality standpoint, the incomplete conversion of wort sugars invariably yields a final product with an altered flavour profile characterised by elevated levels of worty Strecker aldehydes (e.g., methional and 2- and 3-methylbutanal) and a deficiency of the fruity esters and higher alcohols that define the character of conventional lagers [33,50]. Therefore, rapid and accurate analytical monitoring throughout production is essential for these methods, despite the fact that they do not require complex post-fermentation equipment.

Furthermore, from a microbiological safety perspective, the consequences of low or absent ethanol content are substantially more serious than flavour deficiency alone. In conventional beer, microbial stability is maintained by a combination of intrinsic hurdles, such as ethanol (typically 4–10% ABV), iso-α-acids from hops, low pH (3.8–4.5), dissolved CO_2_, limited oxygen, and residual nutrients, which together form a robust barrier against the survival and proliferation of spoilage organisms and pathogens [51]. The removal or significant reduction in ethanol, which is intrinsic to all methods for NABLAB production, substantially erodes this barrier system. The elevated residual sugar content of these beers further exacerbates this risk by providing a nutritional substrate for microbial growth [51].

Two distinct, yet interrelated, microbiological risks must also be considered. The first is secondary fermentation in the final package. Residual fermentable sugars and viable yeast cells or contaminant microorganisms, particularly lactic acid bacteria (LAB) such as *Lactobacillus* and *Pediococcus* species, and wild yeasts such as *Brettanomyces*, can re-initiate fermentation after packaging. This generates CO_2_, causing pressure to build up within sealed bottles and cans. This can result in a loss of container integrity and spoilage [51,52]. The second risk, which is arguably more concerning, is the survival and potential growth of foodborne pathogens. A controlled challenge study demonstrated that *E. coli* O157:H7 and *S. enterica* inoculated at ~10^5^ CFU/mL increased by approximately 2.00 log (to ~10^7^ CFU/mL) in non-alcoholic beer (below 0.5% ABV) held at 14 °C for 63 days. Meanwhile, growth of over 1.00 log was observed in low-alcohol beer (3.2% ABV) under the same conditions. No growth occurred in either beverage type at 4 °C [53]. In view of the low infectious dose reported for *E. coli* O157:H7 in particular, this finding suggests that temperature control throughout the distribution chain is the critical control point for microbiological safety in NABLABs, rather than residual ABV or pH alone. This reinforces the case for validated pasteurisation or sterile filtration, combined with mandatory cold-chain distribution guidance, for these products.

These findings establish that, unlike conventional beers, which are generally considered hostile to pathogenic bacteria, NABs must be treated as food products requiring explicit food safety controls [53]. The authors recommended that the formulation of all NABs with a pH above 4.20 should be reviewed by a relevant authority and that pasteurisation, sterile filtration, and, where appropriate, the addition of approved antimicrobial compounds should be considered mandatory mitigation strategies. Britton and Hill (2025) further support these recommendations in a comprehensive review of microbiological quality control in NoLo beer production, concluding that the gradual phasing out of traditional antimicrobial barriers in NABs requires a fundamental reassessment of the microbial risk framework applied to this product category [54].

In light of the above, thermal pasteurisation, typically applied as tunnel pasteurisation after packaging or flash pasteurisation before filling, remains the most reliable and widely implemented strategy for ensuring the microbiological stability of NABLABs produced by biological pre-processing methods [52,54]. When pasteurisation is incompatible with the product’s sensory profile (e.g., unpasteurised or dry-hopped NABs), sterile membrane filtration coupled with aseptic filling under strictly controlled hygienic conditions is an alternative option. However, this method requires rigorous process validation and continuous environmental monitoring.

#### 4.1.7. Biological Methods: Common Challenges and Future Directions in Research and Development

Although limiting maceration, arrested fermentation, cold contact fermentation, special yeast strains, and yeast immobilisation act on different control points in the beer production process (at the enzymatic, temporal, thermal, genetic, and engineering levels, respectively), they all converge on a common underlying technological cause and, consequently, on a common set of challenges related to quality, safety, and implementation that can be best addressed through an integrative research programme rather than one focused on a specific approach. Firstly, from a sensory and compositional standpoint, it is evident that every biological strategy results in a limitation in ethanol production by reducing the extent or duration of yeast metabolic activity. Irrespective of the specific mechanism, this incomplete or limited fermentation invariably produces a final product characterised by high levels of residual sugars, an excessively sweet flavour, and the accumulation of unmetabolized Strecker aldehydes derived from the wort (e.g., methional, 2-methylbutanal, and 3-methylbutanal), along with a lack of the fruity esters and higher alcohols that define the character of conventional lagers [33,49,50]. This common organoleptic characteristic is not a flaw inherent to any single method. Rather, it is an intrinsic consequence of the very principle of biological dealcoholisation. This explains why rapid and accurate analytical monitoring throughout the entire production process is essential in all these approaches.

Secondly, from a microbiological safety perspective, all biological pre-processing methods substantially erode the combination of intrinsic hurdles that typically protect conventional beer from spoilage organisms and pathogens. Two interrelated risks have been identified in all five approaches: secondary fermentation in the final package, driven by residual fermentable sugars and viable yeast or contaminant microorganisms [51,52], and the survival or growth of food-borne pathogens [53]. The findings establish that, in contrast to conventional beers, NABLABs produced by biological methods must be regarded as food products necessitating explicit, harmonised food safety controls [54], irrespective of the specific biological strategy employed to attain the desired ABV.

Thirdly, all biological routes share a common process-engineering and analytical bottleneck: the majority of literature data are derived from laboratory or pilot-scale trials, and the field still lacks standardised, real-time, in-line analytical tools capable of tracking Strecker aldehyde formation, residual extract, and microbial load consistently across mashing, AF, CCF, special-strain, and immobilised-cell systems. This limits reproducibility and complicates process transfer to industrial scale [1,43].

Addressing these three interconnected challenges, sensory/compositional, microbiological, and process-analytical, rather than optimising each biological method in isolation, represents the most promising route towards the successful industrial implementation of biological dealcoholisation. Future research and development efforts aimed at the effective implementation of biological methods in the brewing industry should focus on aspects such as the rational and combinatorial development of yeasts. This would involve obtaining strains that limit maltose utilisation, maintain or enhance aldehyde reductase activity, and preserve ester formation pathways. This approach would combine the distinct advantages currently observed separately in CCF (aldehyde reduction) and in speciality strains (limited attenuation) [22,23,24,43]. To address the issues of residual sweetness and the aromatic gap, a systematic evaluation of combined strategies is also necessary. Examples of such strategies could include, for instance, the use of special immobilised strains under cold contact conditions or biological pre-treatment combined with minimal, targeted physical finishing. These strategies would aim to improve the flavour profile while preserving the “naturally produced” product positioning so highly valued by craft brewers [17,49]. Another promising area for exploration is the development and validation of in-line or at-line sensors (e.g., electronic “tongue/nose,” rapid chromatographic or spectroscopic methods) combined with predictive process models. This approach would enable real-time monitoring of Strecker aldehydes, residual sugars, and microbial indicators. The result would be a stricter process control and faster product release across all biological methods [1,43]. The design of quantitatively validated combinations of mild heat treatment, approved natural antimicrobials, and innovations in packaging, supported by formal microbial risk assessment frameworks and, where appropriate, a regulatory review of formulations with a pH greater than 4.20 [51,52,53,54], undoubtedly represents one of the industry’s challenges. Finally, a key area for future research is the realisation of systematic studies, from the pilot phase to the industrial phase, that compare biological methods under comparable process and economic conditions. This is because the currently available evidence is largely limited to the laboratory or pilot scale, thereby restricting producers’ technological choices.

### 4.2. Physical Methods

Physical dealcoholisation methods are also known as post-processing techniques. In general, they are deemed to produce an unbalanced beer flavour [44,55], with increased perceived acidity due to the removal of key esters and higher alcohols [56].

However, with advancing technology, there have been occurrences of good efficacy of dealcoholisation and discrete flavour recovery by exploiting a combination of techniques.

Physical methods can be categorised into thermal, membrane separation, and extraction methods.

#### 4.2.1. Thermal Methods

These methods include vacuum distillation, thin-layer evaporation, and spinning cone columns (SCC), which are generally regarded as costly and energy-intensive processes.

(i) *Vacuum Distillation*

Vacuum distillation is a thermal dealcoholisation technique in which beer is processed under sub-atmospheric pressure, exploiting the direct relationship between pressure and boiling point to achieve ethanol separation at temperatures well below those required under atmospheric pressure. In a typical continuous plant configuration, pre-filtered beer is first pre-heated in a plate heat exchanger before entering a distillation column operating at pressures in the range of 60–200 mbar. Under these conditions, the normal boiling point of ethanol (78.4 °C) is reduced to approximately 34–50 °C, depending on the specific operating pressure applied, enabling the stripping of ethanol, CO_2_, and co-distilling volatile compounds from the aqueous beer matrix, with substantially reduced thermal stress on heat-sensitive components [17,57]. The principal limitation of vacuum distillation is the unavoidable co-evaporation of flavour-active volatile compounds alongside ethanol. Key aroma-bearing species, including the esters ethyl acetate, isoamyl acetate, and ethyl caproate, as well as higher alcohols such as isobutanol, 3-methylbutanol, and 2-phenylethanol, exhibit sufficient vapour pressure under vacuum conditions to partition preferentially into the distillate fraction, resulting in a dealcoholised beer that is markedly impoverished in fruity and estery character [33,58,59]. It is often assumed that the extent of aroma loss depends strongly on the operating pressure because lower pressures allow for lower operating temperatures. However, the available simulation evidence does not support the idea that pressure has a substantial effect on ester retention within the operating window normally employed in industry. Horácio and colleagues evaluated three pressures (60, 102 and 200 mbar) in three process configurations: standard distillation (process A); blending with unprocessed beer (process B); and recirculation of the aroma-rich distillate (process C) [57]. They found that the retention of esters and higher alcohols remained essentially constant across pressures within each configuration, indicating that the configuration itself was the determining factor. Ethyl acetate remained below 0.1 µg/L in process A at all three pressures but reached approximately 1.6 µg/L in processes B and C, more than one order of magnitude higher. This behaviour is consistent with the underlying phase equilibria: the principal esters and higher alcohols are more volatile than ethanol in a dilute aqueous solution. Therefore, their relative volatility and the extent of their co-stripping vary only marginally over the 60–200 mbar range. Reducing the operating pressure primarily serves to lower the process temperature, thereby limiting thermal degradation and the development of cooked or caramelised notes rather than preventing the co-evaporation of aroma-active volatiles [17,58,59]. Therefore, aroma recovery and reintegration, rather than pressure optimisation, is an effective method of preserving the aroma profile in vacuum distillation. Consequently, contemporary vacuum distillation systems incorporate an aroma recovery circuit, which is commonly a secondary condenser or a rectification column. In this circuit, the volatiles extracted from the beer are concentrated in a vapour fraction and subsequently re-blended with the de-alcoholised base beer.

From an energy perspective, vacuum distillation is more efficient than atmospheric-pressure distillation, because the reduction in operating pressure reduces the thermal energy input required to drive ethanol evaporation and enables operation at temperatures as low as 34 °C [17,57]. In terms of capital and operational complexity, the technology benefits from relatively straightforward plant engineering and is amenable to integration within existing brewery infrastructure, making it one of the most widely adopted dealcoholisation methods in both the wine and beer industries. Nonetheless, despite these practical advantages, vacuum distillation is less effective than membrane-based methods in terms of aroma compound retention [60].

(ii) *Thin-Layer Evaporation*

In this process, the liquid to be separated passes from a distribution system located in the evaporator head to the heating tubes, flowing downwards in a thin layer and reaching the evaporation temperature at the bottom of the tubes. Evaporation is also promoted by co-current vapour flow [37]. This strategy increases the gas–liquid interphase area and mass transfer into the gas phase, which flows upward after evaporation. Subsequently, vapour separation allows the separation of the dealcoholised beer concentrate from the alcohol-rich vapours, which are then condensed [37]. The residence time in the device is shortened, thereby reducing thermal damage [43]. The fundamental process parameters that can be tuned are the heating steam supply and evaporation temperature, which can be adjusted by vacuum pump control [37].

(iii) *Spinning Cone Columns (SCC)*

**Figure 4:**
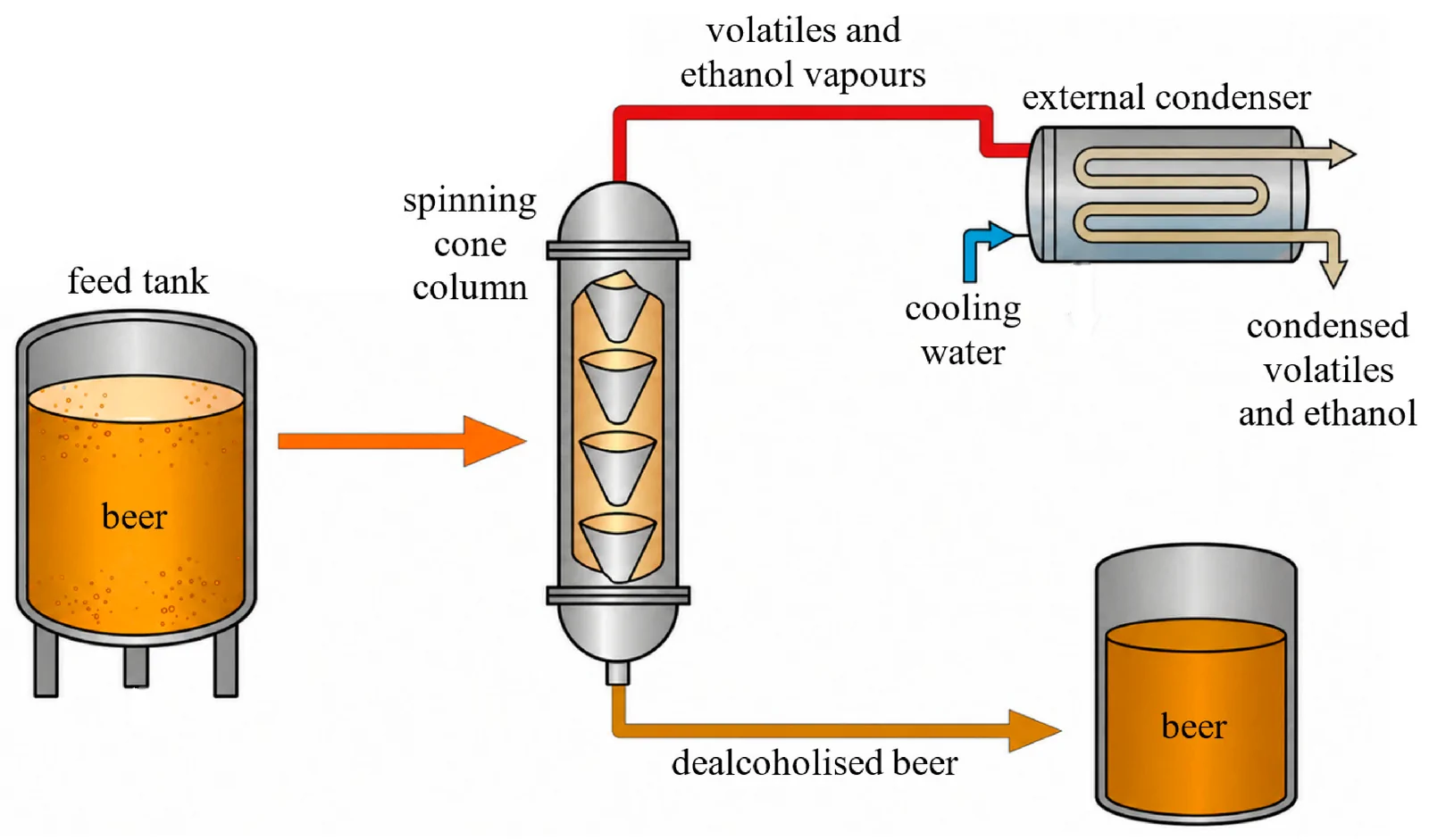
Schematic representation of the thermal dealcoholisation of beer using a spinning cone columns system.

The SCC is a highly efficient and versatile steam-stripping column that uses gentle mechanical forces to maximise the contact between the liquid and vapour phases. The design is intended to facilitate the rapid and efficient separation of volatile compounds, including aroma and alcohol compounds, from thin-film liquid systems. The instrument consists of 1–12 hollow cones operating under vacuum conditions and at low temperatures (35–60 °C). The principle is similar to that of the thin-layer evaporation technique, the difference being that the SCC exploits centrifugal force, in addition to gravity, to form a thin layer of beer to be stripped of ethanol [33,61]. Beer enters the instrument through a feed tube and injection nozzles on the underside of the cone (Figure 4).

As the cone starts spinning, centrifugal force spreads the beer over the entire surface with an approximate thickness of 100 µm, thus promoting ethanol evaporation. The concentrated, dealcoholised beer is collected, while the ethanol and volatiles’ vapours are conveyed to an external condenser for recovery [37]. The heat-sensitive components of beer, as well as its natural colour and aroma, are preserved [61]. From an energy cost perspective, SCC exhibits lower utility consumption in terms of both steam and cooling water requirements compared to alternative thermal dealcoholisation technologies, such as vacuum distillation, nanofiltration-distillation hybrids, and evaporative pervaporation. This efficiency advantage principally arises from two of the SCC’s design features: high mass transfer efficiency, achieved through centrifugal thin-film liquid contact, which maximises the liquid–vapour interfacial area without requiring product recycling, and the absence of external reflux, which eliminates the need for re-evaporation of condensed product, thereby reducing both steam and coolant consumption [61,62].

#### 4.2.2. Membrane Methods

Membrane separation processes are widely recognised for their significant advantages in beverage dealcoholisation. These processes operate at moderate temperatures and low pressures, which help minimise the thermal and chemical stress on the product. This, in turn, contributes to the enhanced preservation of the delicate sensory profile. These methodologies generally demonstrate minimal energy consumption and negligible chemical additive requirements. This characteristic results in a reduction in the detrimental impacts on the product and the potential for reduced operating expenses.

The most commonly used membrane processes for the dealcoholisation of beer are outlined below.

(i) *Reverse osmosis (RO)*

During this process, the beer flows tangentially to the membrane surface. Alcohol, water, aromatics, and carbon dioxide selectively permeate the membrane as the transmembrane pressure exceeds the osmotic pressure of the beer, thereby reducing its volume. Subsequently, diafiltration occurs, wherein demineralised water replaces the previously removed permeate. In the final stage, called the make-up phase, demineralised water is added to the beer to replenish it to its original volume, further reducing the alcohol content. The final product requires re-carbonation [49,63], and Catarino et al. (2007) stated that large molecules (e.g., larger aromatic compounds) are mostly retained on the retentate side of the membrane, even at high pressure and low temperature [64]. From a commercial perspective, RO is a widely used technique in the brewing industry and could play an increasingly important role in the production of NABs. The competitive advantages of this process are attributable to its ability to operate at mild temperatures while maintaining a low energy consumption. Unfortunately, RO becomes progressively less economically feasible when targeting residual alcohol contents below 0.45–0.5% ABV. In fact, as dealcoholisation proceeds, ethanol flux decreases substantially, resulting in longer processing times, higher water consumption and, thus, increased operating costs. Consequently, recent studies indicate that very low alcohol levels are more commonly achieved through hybrid membrane processes than by RO alone [33,65,66]. Moreover, the preliminary installation expenses may be substantial, rendering it a more viable option primarily for leading breweries, with considerable capital investment. Notwithstanding this fact, the technology is scalable and adaptable to different brewery sizes and types.

(ii) *Pervaporation*

This process exploits semipermeable membranes to promote the separation of alcohol from beer in the gas phase, allowing ethanol to selectively migrate to another phase in which it is less concentrated. This is a costly process that requires specialised instrumentation [67]. This technology also allows the extraction and concentration of volatile aroma compounds, which are subsequently added to the final dealcoholised beverage [37].

(iii) *Nanofiltration (NF)*

NF is a pressure-driven membrane separation process that occupies an intermediate position between RO and ultrafiltration in terms of membrane selectivity, operating with a molecular weight cut-off (MWCO) typically in the range of 200–1000 Da. This selectivity range makes NF particularly well-suited for beer dealcoholisation, as ethanol (MW 46 Da) and water (MW 18 Da) pass freely through the membrane, while larger macromolecules responsible for beer body, colour, bitterness, and aroma, including iso-α-acids (MW ~350 Da), polyphenols, proteins, and colloidal compounds, are substantially retained on the retentate side [60,66]. Operationally, NF dealcoholisation follows a two-stage protocol analogous to that used in RO: a concentration phase, in which the beer is progressively dewatered and concentrated by tangential-flow filtration under transmembrane pressures typically in the range of 5–15 bar, is followed by a diafiltration phase. During this step, the alcohol-rich permeate is replaced by deionised or demineralised water fed to the retentate side, progressively diluting and washing residual ethanol out of the concentrated beer matrix until target ABV values below 0.5% (*v*/*v*) are reached [60]. The final product is, then, reconstituted to the original volume, yielding a dealcoholised beer whose real extract, the sum of all non-volatile dissolved solids, is preserved at a level substantially higher than that achievable by thermal methods at comparable alcohol removal efficiencies.

A key advantage of NF over RO is the lower operating pressure required to drive adequate ethanol flux through the membrane, reducing both the energy consumption per unit volume processed and the mechanical stress imposed on the beer matrix. Bóna et al. (2023) demonstrated that pilot-scale dealcoholisation of both filtered and unfiltered lager beer using hollow-fibre polyelectrolyte multilayer (PEM) NF membranes, with a MWCO of approximately 400 Da, was achievable at transmembrane pressures of 5–8.6 bar, with a permeate flux of 10 L m^−2^ h^−1^, yielding near-complete ethanol passage (~100% passage) alongside a real extract loss of only 15–18% [60]. These authors further reported that the dealcoholised product received an acceptable sensory evaluation, which could be significantly improved by restoring the minerals and glycerol lost through the membrane during the diafiltration step, highlighting that ion and small-molecule management represent critical quality control parameters in NF-based processes [60]. NF is also distinguished from RO by its larger-pore membranes, which allow inorganic ions (such as Na^+^, K^+^ and Cl^−^) to pass through relatively easily. This can alter the ionic balance and, consequently, the mouthfeel and perceived bitterness of the final product. Therefore, it must be carefully accounted for in process design [60,66].

A further technically and economically relevant application of NF in NABLAB production is its integration into hybrid process configurations. Sánchez and colleagues conducted a conceptual design study of a hybrid NF/distillation process to produce alcohol-free beer [68]. In this process, the ethanol-water permeate from the NF stage was upgraded on site through rectification to produce a high-strength spirit suitable for gin production, converting a waste stream into a revenue-generating co-product. For an annual production capacity of 720,000 litres, the hybrid process incurred operating costs only 6.2% higher than a standalone NF process (USD 205,500 versus 193,600 per year), while delivering a substantially lower environmental footprint as assessed by life cycle analysis [66,68]. This hybrid approach demonstrates the potential of NF as a foundation technology for the production of circular and economically viable NABLAB processes. Challenges that remain to be addressed at an industrial scale include membrane fouling by colloidal polysaccharides, β-glucans, proteins, and polyphenols, which are intrinsic to the beer matrix. These compounds progressively reduce permeate flux and may require periodic chemical cleaning, which affects both membrane longevity and product quality [60,66].

(iv) *Osmotic distillation (OD)*

The utilisation of OD has emerged as a particularly promising membrane-based technology for the production of NABLABs. OD, also known as membrane evaporation, isothermal membrane distillation or osmotic evaporation, is a separation process in which a liquid feed containing a volatile component is put in contact with a hydrophobic microporous membrane whose opposite face is exposed to a second liquid phase capable of absorbing that component [69]. In the context of beer dealcoholisation, the ethanol transport mechanism unfolds in three sequential steps: (i) evaporation of ethanol at the membrane pores on the feed side, (ii) diffusion of the ethanol vapour through the gas-filled membrane pores, and (iii) condensation at the stripping solution interface on the permeate side. The overall driving force is the difference in ethanol vapour pressure across the membrane [70]. In the case of the microporous membranes that are generally employed, operating at ambient temperature and atmospheric pressure, both Knudsen diffusion and molecular diffusion are contributing factors to mass transfer. The relative contribution of these factors is governed by the pore diameter: molecular diffusion increases and Knudsen diffusion decreases as the pore diameter enlarges [71].

From a thermodynamic perspective, a significant benefit of OD compared to conventional thermal dealcoholisation techniques (e.g., vacuum distillation or falling-film evaporation) is its isothermal nature. Membrane processes, such as OD, protect volatile compounds from thermal damage while exhibiting low energy consumption, making them particularly well-suited for delicate matrices such as craft beers [72]. Phenolic compounds, due to their high molecular weight, do not permeate the membrane in vapour phase and therefore undergo no significant change during OD treatment [71]. In a similar manner, carboxylic acids, being more polar than ethanol, exhibit limited transport through hydrophobic polypropylene membranes, so their composition remains largely unaltered, with the notable exception of acetic acid, whose higher volatility drives selective co-permeation [73]. Nevertheless, OD is not without its limitations. It has been reported that the process causes a loss of volatile compounds, mainly esters and higher alcohols, which are considered key contributors to beer aroma and taste [74]. Indeed, in the 2015 study by Liguori and colleagues [70], an alcohol-free beer (<0.5% *v*/*v* ethanol) was produced from a conventional beer by a modified osmotic distillation configuration operating at ambient temperature and atmospheric pressure. In this configuration, the aqueous stripping solution was recycled from one batch to subsequent batches in order to reduce water consumption and operating costs. In these specific conditions, and relative to the original beer, the losses amounted to approximately 77% for higher alcohols, 99% for esters, and 93% for aldehydes. It is imperative to note that these figures pertain to a single beer and a specific combination of membrane-contactor configuration, degree of dealcoholisation and stripping-solution regime. Consequently, they should not be extrapolated to osmotic distillation in general. The extent of volatile loss is contingent on the ethanol removal target, the membrane area-to-feed volume ratio, the composition and renewal rate of the stripping phase, and the beer matrix itself. The same research group reported that the stripping solution, prepared by diluting the original beer with water rather than using water alone, led to significantly enhanced retention of aldehydes and 2-phenylethanol in top-fermented beers [75]. Furthermore, they observed that malt-forward styles, such as stouts, exhibited a sensory profile that more closely resembled the original product compared to pale lagers [74]. The values reported above should therefore be interpreted as indicative of the upper limit of volatile loss achievable when OD is employed to achieve complete dealcoholisation with an aqueous stripping phase, rather than as a general performance metric of the technique. Nevertheless, they are comparable to the reported losses for dialysis, falling-film evaporation, vacuum distillation and reverse osmosis operated to the same ethanol endpoint [70], which is the substantive point for the purposes of the present comparison.

From an economic standpoint, a cost appraisal of OD-based dealcoholisation has shown that operating costs are disproportionately driven by the consumption and cost of the stripping water, a significant drawback for industrial-scale implementation [76]. Furthermore, in 2021 De Francesco and colleagues observed that the characteristic dryness typical of certain types of beer appears to be less suitable to OD than beers with a pronounced malty character [74]. Malt-forward beers, such as stouts, exhibit sensory profiles after dealcoholisation that closely resemble those of their regular counterparts, while pale lager is more adversely affected by the treatment [74].

In order to overcome the incomplete dealcoholisation achievable by OD alone, recent studies have explored hybrid approaches. These include membrane OD applied for partial dealcoholisation of beer, followed by hydrophobic–hydrophilic pervaporation with zeolite membranes. This allows the production of low-alcohol beer with approximately 2.5% *v*/*v* ethanol, achieving a compromise between low alcoholic degree and preserved sensory properties [73]. Recently, Arundhathi and colleagues explored the utilisation of in-house-fabricated PVDF flat-sheet membranes with NaCl draw solutions, demonstrating the viability of OD for partial ethanol removal at ambient temperature and zero transmembrane pressure, thus expanding the technological repertoire available for sustainable dealcoholisation [77].

(v) *Dialysis*

Ethanol removal via dialysis is one of the earliest ways of dealcoholising beer using a membrane, and it was first shown to work on a small scale in 1982 by Moonen and Niefind [78]. In this process, a semi-permeable membrane is employed to separate the beer and the dialysate (aqueous solution) flowing in a countercurrent. This enables operation at very low temperatures, typically between 1 °C and 6 °C, thereby minimising thermal damage to heat-sensitive beer constituents [79].

Substance exchange occurs almost exclusively via diffusion phenomena [75], and the degree of fraction exchange depends on the concentration gradient and contact time. Generally, porous hydrophilic membranes of Cuprophane are the most widely used material for dialysis-based beverage dealcoholisation [80]. Dialysis has been shown to offer a principal thermodynamic advantage over conventional thermal dealcoholisation techniques, residing in its isothermal and isobaric character, due firstly to the absence of phase transition, and secondly to the low operating temperatures. The combination of these factors preserves the chemical integrity of thermolabile aromatic compounds, while minimising energy consumption [79]. Furthermore, when transmembrane pressure is controlled below critical thresholds, the concentration-driven nature of dialysis minimises the loss of high-molecular-weight beer constituents, such as proteins, polyphenols, and polysaccharides, which are primary contributors to body, mouthfeel, and colloidal stability of beer [81]. From an operational standpoint, dialysis systems do not require elevated pressures, unlike RO, reducing mechanical complexity and extending membrane service life, while retaining CO_2_ within the system when appropriately designed [48]. However, the degree of alcohol reduction is directly proportional to the dialysate flow rate: an increase in dialysate flow rate, whilst enhancing dealcoholisation yield, concomitantly results in a significant reduction in body in the treated beer. This indicates a fundamental conflict between dealcoholisation efficiency and the preservation of sensory quality [79]. This conflict stems from the inherently limited selectivity of dialysis, which is generally insufficient to separate ethanol from beer without simultaneously extracting other low-molecular-weight aromatic compounds, including key aromatic esters and higher alcohols. Added to this is the dilution caused by the transfer of water from the dialysate to the beer, driven by the osmotic gradient across the membrane, leading to a general loss of aromatic integrity in the final product [48]. Although dialysis is a mature and historically significant technology, it has therefore been gradually replaced in new industrial plants by RO and NF, which offer greater selectivity with regard to ethanol and the possibility of achieving very low levels of residual alcohol [80].

#### 4.2.3. Extraction Techniques

The available literature on extraction techniques for beer dealcoholisation is limited. The main findings are as follows.

- *Solvent extraction*

This process exploits the different solubility of ethanol and water in other solvents, but tends to strip flavours, and traces of solvents are often left in the matrix [43]. Suitable solvents that have been indicated for such an application are propane, polypropylene, and butane; using them, ethanol can be concentrated up to 96% in weight. Azeotropic distillation with these solvents can be used to separate ethanol from water [82]. One solvent that could be particularly promising for this type of approach is supercritical carbon dioxide, which has already been used in the dealcoholisation of wines, but has yet to be thoroughly investigated in the brewing industry [54].

**Figure 5:**
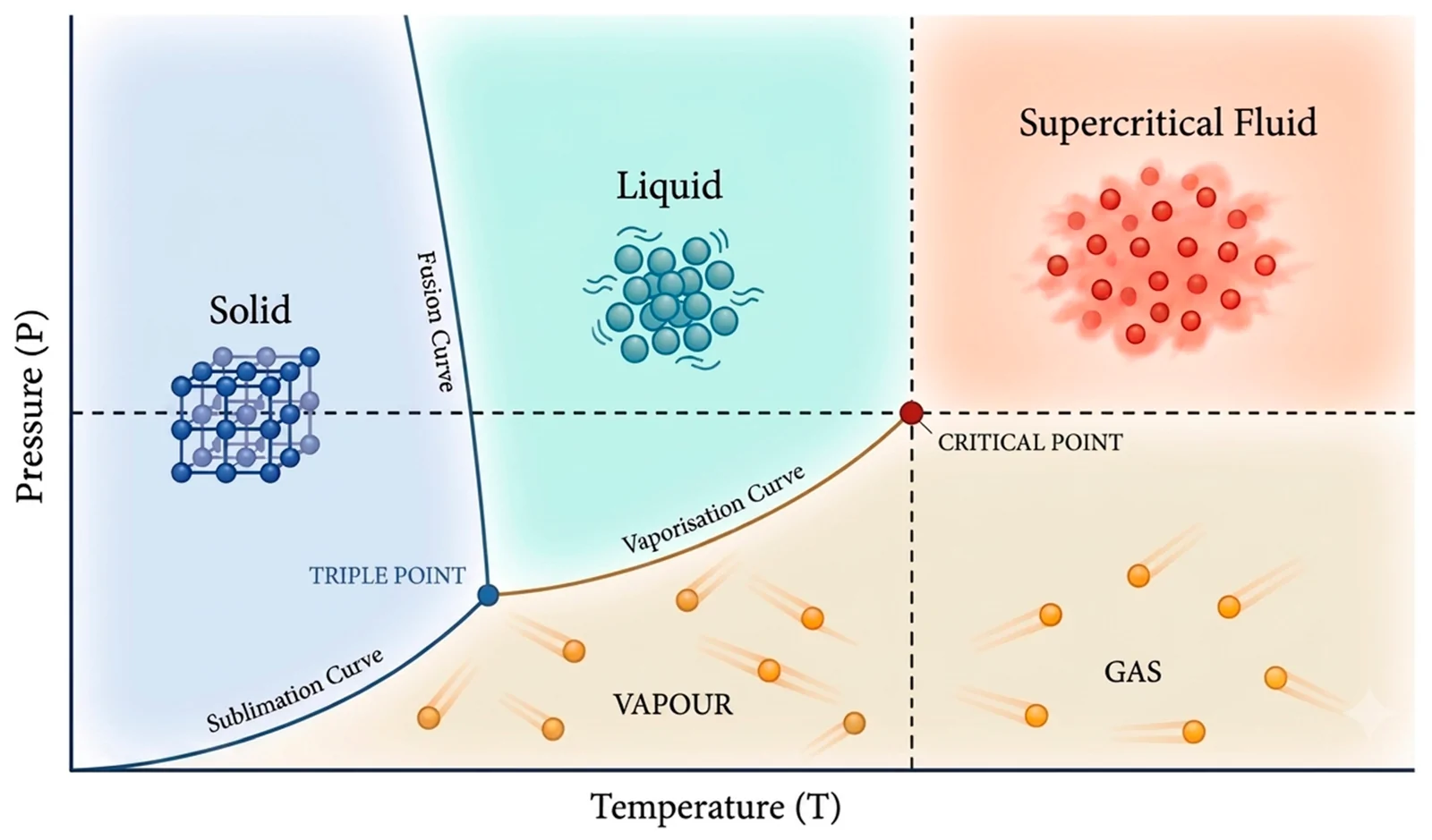
Representative phase diagram illustrating the physical states of matter as a function of pressure (P) and temperature (T). The diagram highlights the phase boundaries between solid, liquid, vapour, and gas, the triple point, and the critical point leading to the formation of a supercritical fluid phase.

Near the critical point of a substance, its isothermal compressibility tends to infinity, and its density changes significantly. In this critical region, gases exhibit liquid-like densities and increased pressure-dependent solvent capacities, which can be used to devise separation processes (Figure 5).

Supercritical fluids can separate the components of a mixture by exploiting the differences in their specific interactions with each component. The critical temperatures of substances differ significantly; hence, the extraction fluid can be chosen depending on the limits and parameters of the target molecules. Carbon dioxide and other hydrocarbon solvents are particularly useful for preserving heat-sensitive analytes because their critical temperatures are near the ambient temperature. By tuning the operative pressure and temperature, the density can be increased, thus affecting the extraction power of the fluid by increasing the probability of interactions between the solvent and solute. Supercritical CO_2_ (SC-CO_2_; critical temperature 31.1 °C, critical pressure 7.38 MPa) extraction has been investigated as a dealcoholisation technique for fermented beverages, and its application to wine and cider has been demonstrated at both laboratory and pilot scales. In these systems, a two-step countercurrent extraction process has been proposed. In the first step, the aroma fraction is selectively recovered from the beverage at low CO_2_-to-feed ratios, and in the second step, the aroma-depleted beverage is dealcoholised at higher CO_2_-to-feed ratios, yielding a product with ethanol content below 1% (*v*/*v*), while retaining antioxidant capacity comparable to the original beverage [83,84]. A key advantage of SC-CO_2_ over thermal dealcoholisation methods is its selectivity: water, proteins, carbohydrates, and non-volatile matrix components are not substantially removed or denatured under supercritical conditions, since CO_2_ in its supercritical state acts as a tuneable solvent whose density, and hence solvation power, can be adjusted by varying operating pressure and temperature [33,83]. However, it must be clearly stated that, to date, SC-CO_2_ dealcoholisation has not been implemented at a commercial scale for the production of NABLAB, nor has it been adopted as a standard industrial dealcoholisation process for any beverage category. As a matter of fact, this technology remains at a pre-commercial stage for beer, owing to a combination of barriers that significantly hinder its industrial translation. The requirement for high-pressure equipment operating in the range of 9–18 MPa entails substantial capital investment and stringent engineering safety requirements. The high CO_2_-to-feed mass ratios necessary to achieve ethanol reduction to below 0.5% ABV (typically >30 kg CO_2_/kg feed) render the process energy-intensive at scale. The co-extraction of volatile aroma compounds alongside ethanol, while manageable through the two-step design, remains a technical challenge that has not yet been fully resolved for the compositionally more complex beer matrix [33,54]. Therefore, SC-CO_2_ dealcoholisation of beer should be regarded as a promising but still emerging technology, requiring further research and process optimisation before it can be considered a viable alternative to established methods such as vacuum distillation, spinning cone columns, or membrane-based dealcoholisation.

ii. *Adsorption*

Adsorption is a less commonly used process for ethanol removal in the production of NABs. It consists of using an adsorbent phase with a high affinity for ethanol, typically zeolites, resins, or kieselgels, which can selectively entrap the alcohol [49,85]. The process involves separating the original beer into an aqueous stream (alcohol-free beer) and an alcoholic adsorbed phase. Following the removal of ethanol, the adsorbent is regenerated using a gaseous stream such as carbon dioxide. However, this approach is not without drawbacks: (i) conventional adsorbents are also capable of extracting other beer aroma compounds; (ii) to dealcoholise the desorbed phase, it is necessary to install an additional distillation column; (iii) the process is costly and only suitable for specific applications.

### 4.3. Comparative Analysis of Dealcoholisation Methods

For beer producers, selecting the most suitable dealcoholisation method is a complex decision that requires careful consideration of various factors. Table 3 provides a comprehensive comparison of the key performance indicators, serving as a multicriteria decision matrix. A methodical presentation of data on alcohol removal efficiency, impact on flavour, energy consumption and market acceptance can assist brewers in conducting a thorough evaluation of each method. This approach enables them to identify areas of strength and weakness, as well as assess the suitability of each method for specific needs. For instance, a method might excel at removing alcohol, but falter in preserving flavour, or it might offer low energy consumption, but require a substantial initial investment [43,49,61,71,86,87]. However, beyond this method-by-method comparison, the thermal, membrane, extraction and biological routes described above share a small number of underlying technological trade-offs that recur across categories (Table 3).

#### 4.3.1. The Selectivity–Aroma Retention Trade-Off

The most common issue affecting almost all dealcoholisation processes is the similarity between ethanol and the volatile compounds that define beer aroma. Esters (e.g., ethyl acetate, isoamyl acetate), higher alcohols (e.g., isobutanol, 3-methylbutanol, 2-phenylethanol), and Strecker aldehydes share molecular weight, polarity and vapour pressure with ethanol. As a result, any separation process driven by volatility, diffusivity or membrane permeability inevitably co-removes a substantial fraction of these desirable compounds alongside the target alcohol. Biological methods avoid this issue by not involving any separation steps, but they have an analogous compositional deficit. Since fermentation is curtailed or restricted rather than completed, the same classes of desirable esters and higher alcohols are never synthesised in sufficient quantity. As a result, unmetabolized wort-derived Strecker aldehydes accumulate, producing a comparable net sensory gap relative to conventional beer [33,49,50]. In both cases, therefore, the sector faces essentially the same fundamental challenge: reducing the alcohol content without proportionally diminishing the product’s aromatic profile, even though the physical or biological mechanism underlying this discrepancy is different. The industry’s primary technical response to this shared trade-off has been the recovery and reconstitution of aroma, involving the re-condensation and re-blending of the volatile fraction removed during vacuum distillation [57], the recovery of aroma compounds via pervaporation for reincorporation into the dealcoholised base beer [62,67], or the coupling of osmotic distillation with pervaporation to partially rebalance the composition [73]. While these hybrid aroma-recovery strategies are technically feasible and measurably improve sensory outcomes, none has yet demonstrated full parity with the aroma profile of conventionally fermented beer. Furthermore, their added process complexity constitutes a barrier to industrial adoption, particularly for small and medium-sized breweries.

#### 4.3.2. Energy, Capital Investments, and Scalability

A second recurring trade-off concerns balancing energy demand, capital cost and scalability. The energy efficiency of thermal methods varies considerably: vacuum distillation is comparatively favourable because reduced operating pressure lowers the temperature, and thus the energy required to evaporate ethanol [17,57], while spinning cone columns further reduce utility consumption thanks to their high mass-transfer efficiency and lack of external reflux [61,62]. Membrane processes generally operate at ambient or moderate temperatures, which results in lower thermal energy demand. However, this advantage is often offset by the need for high-pressure equipment (e.g., in RO and NF), high capital expenditure, and operational costs associated with membrane replacement and cleaning. This can make these technologies primarily economically viable for larger breweries with the necessary capital base [60,61,66]. The trade-off between low energy consumption but a high initial investment and higher energy consumption but lower initial costs means that the choice between the thermal and membrane processes often depends as much on a brewery’s financial profile and production scale as on its sensory objectives for the finished product.

Extraction-based methods represent the opposite extreme. Supercritical CO_2_ extraction offers excellent selectivity and preserves non-volatile matrix components. However, its industrial implementation is hindered by the high capital costs of the necessary high-pressure equipment (9–18 MPa), the extremely high CO_2_-to-feed mass ratios required (typically >30 kg of CO_2_ per kg of beer) and the issue of co-extraction of aroma compounds in more complex beer matrices. This means that, despite its established use in wine de-alcoholisation, it has not been implemented on a commercial scale for beer yet [33,54,83,84]. Biological methods are at the opposite end of the capital investment spectrum. They require no dedicated post-fermentation equipment and can be implemented within existing brewery infrastructure. This explains their popularity among craft producers. However, this operational simplicity is offset by a comparatively narrow processing window and a greater reliance on strain or process expertise to control off-flavour formation. There are also additional downstream requirements, such as pasteurisation or sterile filtration, to ensure microbiological safety [49,51,52,53,54]. A related and frequently overlooked scalability bottleneck is technology readiness. Although vacuum distillation, spinning cone columns, and reverse osmosis are well-established in the wine and beer industries, several promising alternatives, such as hybrid nanofiltration/distillation configurations, zeolite-assisted pervaporation coupled with osmotic distillation and supercritical CO_2_ extraction, remain confined to laboratory or pilot-scale demonstration. This creates a two-tier industrial landscape, where the most effective methods in terms of aroma retention or environmental impact are often the least accessible to producers seeking reliable technology, which slows the adoption of innovations in commercial practice.

#### 4.3.3. The Convergence of Microbiological and Compositional Stability as a Final Endpoint

Regardless of the technological route used to reach the target ABV %, both physically and biologically de-alcoholised NABLABs face the same downstream challenge: the substantially reduced ethanol content erodes the antimicrobial barrier that protects conventional beer. This necessitates equivalent post-production safeguards, such as pasteurisation or sterile membrane filtration, across essentially all production routes [51,52,53,54]. This convergence suggests that microbiological stabilisation should be considered a common requirement of NABLAB manufacturing, rather than a limitation of a specific dealcoholisation technology, and underscores the need for standardised, cross-cutting benchmarks for quality and safety in future research and regulatory activities.

### 4.4. NABLAB: Food Safety Concerns

The microbiological considerations set out in Section 4.1.6 do not apply only to biologically pre-processed beers. Erosion of the ethanol hurdle is a common feature of every NABLAB production route. Physically de-alcoholised products are also exposed to an additional, partly distinct set of hazards arising downstream of the separation step:

(i) *Post-process contamination and membrane hygiene*

The dealcoholisation unit is performed after boiling and fermentation and therefore represents a potential point of recontamination. Membrane systems present particular challenges in this regard: RO, NF and OD modules operate at or near room temperature with a nutrient-rich feed stream, making them inherently prone to fouling by polysaccharides and polyphenols [66]. These substances then provide a substrate for biofilm growth. Data from membrane systems in the food industry demonstrate that such biofilms are not necessarily removed by standard clean-in-place (CIP) regimens [89]. Therefore, membrane hygiene, CIP validation and module integrity should be considered critical control points for beer physical dealcoholisation. Similar considerations apply to SCC and thin-film evaporators, whose distributors and internal components are more difficult to clean and validate for sterility than closed batch tanks.

(ii) *The adequacy of the thermal treatment*

Pasteurisation regimes for this product category must be validated rather than transferred from those used for conventional beer. While the recommended regimes for NAB (approximately 73.9 °C for 1 min or 80–120 pasteurisation units) are effective, the reduced regimes that producers may be tempted to adopt to protect the sensory profile of a de-alcoholised product are not. It has been reported that treatment at 52 °C for 1 min (approximately 0.75 PU) reduced *Salmonella enterica* subsp. *enterica* serovar Tennessee by only around 2.2 log CFU/mL in NAB. The organism persisted for at least 183 days at 14 °C, regardless of co-inoculation with the spoilage organism *Levilactobacillus brevis*. By contrast, the standard regime achieved a reduction of around 8 log CFU/mL [90]. Given that the physical dealcoholisation process is often employed precisely to preserve aroma, the incentive to under-pasteurise is greatest in areas where intrinsic antimicrobial hurdles are weakest.

(iii) *Package composition and aseptic filling*

The residual risk in the finished product is governed by its composition and its packaging environment, rather than by the production route. A comprehensive screening of 50 NoLo beers available on the United Kingdom market detected no instances of *Salmonella*, *Escherichia coli*, *Enterobacteriaceae*, *Bacillus cereus* or sulphite-reducing clostridia. In the accompanying challenge tests, no growth of these pathogens was observed at pH 3.8. However, *E. coli* O157 grew only at pH 4.6, and *Salmonella Enteritidis* grew at pH 4.2–4.6, but only when carbon dioxide was reduced and oxygen was elevated. In both cases, bitterness had no effect [91]. Results highlighted that carbonation and oxygen control at filling, together with hygienic or aseptic filling conditions, constitute a genuine hurdle in their own right and should be specified as such.

(iv) *Post-packaging handling and draught dispense*

It is important to note that the hazard does not end at the filler. In a survey of 53 draught NoLo samples, covering 17 brands and seven styles, collected from 12 different pubs in Nottingham, United Kingdom, 54.7% were found to be of unacceptable microbiological quality. The presence of unacceptable samples was positively correlated with high present gravity and high pH. It was also found that microbial activity in the dispensing line raised the ethanol concentration of some samples above the legal limit for the NoLo category in the United Kingdom. This converts a hygiene failure into a labelling non-compliance [92]. Earlier challenge testing had already shown that NoLo beers spoil two- to five-fold more readily than 4.5% ABV controls, and that re-addition of ethanol only produced modest inhibition. It was therefore recommended that these products should not be dispensed through conventional long-line draught systems but require dedicated, hygienically designed equipment [93].

Based on this evidence, a set of technology-neutral controls can be proposed as the minimum requirement for this category, regardless of whether the ethanol was biologically limited or physically removed: (i) formulate to a pH of less than 4.0 and minimise residual fermentable sugars wherever the style permits [92]; (ii) validate a defined thermal treatment or sterile filtration process for the specific product and verify it, rather than assuming transferability from conventional beer [54,90]; (iii) treat the dealcoholisation unit, its CIP regime and the filler as critical control points and perform environmental monitoring, as well as periodic module autopsy for membrane systems [89]; (iv) maintain carbonation and minimise dissolved and headspace oxygen during filling [91]; (v) specify cold-chain distribution since storage temperature, rather than residual ABV or pH alone, has emerged as the dominant determinant of pathogen behaviour [53]; and (vi) use dedicated, hygienically designed dispensing equipment for draught formats [93]. These measures address the progressive removal of traditional antimicrobial barriers in NABLABs, which requires a fundamental reassessment of the microbial risk framework applied to this product category [51,54].

## 5. NABLABs: Production Costs and Environmental Impacts

As highlighted in the previous paragraphs, many dealcoholisation methods are costly and require additional equipment compared to traditional brewing [63,94], an already expensive process *per se*. This is especially true for post-processing methods [56,95,96]. Apart from requiring specific machinery, they are also very energy-consuming, and hence not always convenient or economically applicable to the production of NABLABs, especially at the craft level [33,61].

In this regard, de Bont’s team conducted an analysis of the economic viability of producing alcohol-free craft beer in the Netherlands [97]. The analysis concluded that it is not economically viable for an average Dutch craft brewery to invest in the production of alcohol-free beer, due to the high costs and low production volumes. The principal conclusions of the research project are the following: (i) a minimum production level of 400 hL of alcohol-free beer is required to counterbalance the elevated cost price, and (ii) the production of 250 hL of alcohol-free beer per year can yield a profit of 3 million euros over a 30-year period. However, this is not more than what could be earned by selling alcoholic beer [97].

Although economic feasibility has received more attention in the NABLAB literature, the environmental footprint of dealcoholisation is equally important for the long-term sustainability and industrial acceptability of these products. It also varies considerably depending on whether a biological or physical production strategy is used. A comprehensive assessment must consider at least four dimensions: energy intensity and associated greenhouse gas emissions, water consumption, waste generation, and the potential for by-product valorisation, as well as the additional downstream environmental burden common to all NABLABs, regardless of the dealcoholisation route used. Energy demand differs markedly between different categories of alcohol removal and is a primary determinant of their associated carbon footprint. Although operating under vacuum reduces the temperature and therefore the energy required to evaporate ethanol relative to atmospheric distillation, thermal methods are generally the most energy-intensive [17,57]. This is because vacuum distillation, thin-layer evaporation and SCC still rely on continuous heating, cooling and vacuum generation. These are all electricity- or steam-intensive unit operations, and the associated emissions depend heavily on the carbon intensity of the local energy mix. Membrane-based methods (e.g., RO, NF, OD and dialysis) are generally considered to be lower-energy alternatives since they operate at ambient or moderate temperatures without a phase change [61,79]. However, this advantage may be offset by the energy required to generate and maintain transmembrane pressure in RO and NF. In the case of OD, the disproportionate contribution of stripping water consumption and regeneration to overall operating costs and, indirectly, energy demand, may also offset this advantage [76]. Extraction-based methods occupy the opposite extreme: supercritical CO_2_ de-alcoholisation requires high-pressure conditions to be maintained and very large CO_2_-to-feed mass ratios to be processed, both of which imply a substantial energy and infrastructure footprint. This currently limits its environmental and economic competitiveness at an industrial scale. Biological pre-processing methods avoid the need for energy inputs during post-fermentation, since they achieve dealcoholisation within the existing fermentation infrastructure. However, several of these methods, particularly CCF and dialysis-adjacent cold processing, require maintaining conditions close to 0 °C for extended periods of time, which introduces a refrigeration-related energy demand that is rarely quantified or compared on a like-for-like basis with the heating-related energy demand of thermal physical methods. Therefore, carbon footprint comparisons cannot be inferred from processing temperature alone and require dedicated, method-specific life-cycle inventories.

Another factor of paramount importance in terms of environmental impact is water consumption. Beer production, in fact, is already a water-intensive process, and the additional water burden of several dealcoholisation technologies is often overlooked relative to their energy footprint. RO and NF require a diafiltration step, where the permeate volume removed is replaced with demineralised or deionised water. This is followed by a make-up phase that restores the original beer volume. Therefore, water consumption scales with the targeted degree of alcohol reduction [49,60,63]. OD depends on a hypertonic stripping solution that is continuously regenerated. The consumption and regeneration of this stripping water have been identified as a disproportionately large contributor to the operating costs and, by extension, the resource footprint of the process [74]. Dialysis relies on a continuous counter-current dialysate flow, and the degree of ethanol removal is directly proportional to the dialysate flow rate. This suggests that higher performance in dialysis is intrinsically linked to higher water throughput [79]. As both water and energy consumption tend to increase in proportion to the intensity of ethanol removal in membrane-based processes, water use should be treated as a key environmental indicator alongside energy demand, rather than a secondary consideration, in any comparative assessment of dealcoholisation technologies.

On the other hand, some researchers are focusing on making NABLAB brewing more sustainable by employing the byproducts of the process. For example, Sánchez and colleagues [68] engineered a hybrid nanofiltration/distillation process to produce alcohol-free beer and valorised the byproducts of dealcoholisation by exploiting the recovered ethanol-water mixture for the on-site production of gin. They also performed a cost analysis and assessed the environmental impact of the system [68]. This strategy implied a decrease in the required bioethanol and deionised water for gin production, and savings amounting to up to 26,700 USD/year for the best-case scenario (13.7% of the total costs). Furthermore, the savings in raw materials and the environmental impact equalled the on-site production of 175,000 L per year of this beverage; additionally, the biological treatment of the byproduct was avoided, resulting in lower methane and CO_2_ emissions for dealcoholisation [68].

Despite this virtuous example of byproduct valorisation, the NABLAB industry needs more propositions and efforts towards sustainability and circularity.

Inspiration could be drawn from other industries, such as low-alcohol wines. Esteras-Saz and colleagues proposed the application of OD to partially dealcoholise red wine and then exploit pervaporation on the wastewater from this process [98]. This second step allowed for both the production of second-generation bioethanol and water recycling for another round of OD, yielding similar results to those of the first step [98].

Other strategies have been proposed to make beer production greener and more environmentally friendly, that do not directly concern the production method as much as they affect the employed raw materials. In fact, NABLABs can be produced using low-sugar wort obtained from brewer’s spent grains (BSG). In this regard, Canonico’s team tested fourteen yeast strains from seven species to produce NoLo beers, using wort from BSG [99]. These are constituted by the peel, pericarp, and seeds, with residual amounts of endosperm and aleurone from barley. The exploitation of such wort was bound to yield low alcohol percentages due to its reduced sugar content, while the chosen yeasts could provide probiotic functionalities. The obtained beers were appreciated for their sensory profiles, and five non-conventional brewing yeasts were selected as single-starter inoculums to produce NABLABs [99].

Taken together, the reviewed evidence indicates that a comprehensive environmental assessment of NABLAB production cannot be based on individual, technology-specific case studies. Currently, there is a lack of standardised cradle-to-gate life cycle assessments (LCAs) in the literature that are conducted according to a common functional unit (e.g., per hectolitre of finished NABLAB) and system boundary. These assessments would directly compare thermal, membrane, extraction-based and biological de-alcoholisation routes under equivalent conditions. Such LCAs would require the four dimensions discussed above to be integrated into a single, standardised framework: process energy demand and its associated carbon intensity; direct and indirect water consumption; waste generation and by-product valorisation potential; and the downstream stabilisation burden common to all routes, together with upstream raw-material sourcing strategies [99].

## 6. Conclusions and Future Perspectives

The NABLAB sector is experiencing a significant and transformative shift in the global beverage market. This is driven by an evolving consumer focus on health, changing regulatory frameworks, and innovations in production technology. Despite rapid market expansion and promising economic forecasts, the regulatory landscape is fragmented. Therefore, harmonised definitions and standards are needed to facilitate international trade and provide consumers with clarity.

From a production standpoint, both biological and physical dealcoholisation methods have distinct advantages and limitations that must be considered. Biological methods are highly effective in limiting ethanol formation, but they can also result in sensory challenges, such as residual sweetness and elevated off-flavours, as well as increased microbiological risks due to the diminished antimicrobial barriers. Physical methods, including thermal and membrane-based techniques, offer improve ethanol removal and aroma retention; however, they can be energy-intensive and costly. Emerging technologies, such as SC-CO_2_ extraction, show promise but require further research and industrial validation.

In this context, it is imperative to prioritise food safety considerations, as NABLABs with low ethanol content and high pH values are more susceptible to microbial spoilage and pathogen survival. This necessitates the implementation of rigorous control strategies, including pasteurisation or sterile filtration. Sensory quality remains a critical bottleneck, with the need to balance ethanol reduction and flavour preservation driving ongoing innovation in yeast strain selection, fermentation management, and post-processing techniques.

Producers can reduce their ecological footprint while maintaining product quality by integrating sustainable sourcing of raw materials, energy-efficient processing and biodegradable packaging. In this regard, the valorisation of byproducts, such as the reuse of ethanol-rich streams for spirit production or the use of BSG to generate low-sugar wort, offers promising avenues to reduce environmental impact and improve economic viability. It is recommended that future research efforts concentrate on the optimisation of these integrated processes and the exploration of novel raw materials and microbial strains that offer functional benefits. The successful scale-up and commercialisation of NABLABs, especially at the craft level, is contingent upon multi-criteria decision-making that integrates sensory quality, safety, regulatory compliance, environmental sustainability, and economic feasibility. In order to address the current bottlenecks and realise the full potential of this growing market segment, there is a necessity for collaborative efforts among industry stakeholders, researchers, and policymakers.

## Figures and Tables

**Table 1:** Forecast of NABLAB’s share of total beer sales in major consumer countries (2023–2029, value in %).

Countries	2023	2024	2025	2026	2027	2028	2029
Brazil	1.1	1.1	1.1	1.1	1.1	1.1	1.1
Czech Republic	8.2	8.5	8.7	8.8	8.9	8.9	8.9
France	5.3	5.6	5.8	5.9	6.0	6.1	6.2
Germany	14.2	14.3	14.4	14.5	14.5	14.5	14.6
Japan	6.2	6.5	6.7	6.9	7.0	7.1	7.2
Poland	5.0	5.0	4.9	4.9	5.0	5.0	5.1
Spain	6.7	7.3	7.5	7.7	8.0	8.1	8.2
UK	1.5	1.7	1.8	1.8	1.9	1.9	2.0
USA	0.9	1.0	1.0	1.1	1.1	1.1	1.2

Note: measured as a share of total beer sales volume (thousands of hectolitres). Source: Authors’ elaboration based on data reported by Wein (2025) [40].

**Table 2:** Scenario analysis of the impact of selected restrictions on the beer market over time.

Scenario	% Impact on CAGR Forecast	Geographical Relevance	Estimated Timeframe of Impact
Government regulations	−0.80	Global, but with significant impact in Europe and North America	Medium term (2–4 years)
Consumers’ preference for non-alcoholic or low-alcohol products	−0.60	Global, with early adoption in Europe and North America	Long term (≥4 years)
Increase in raw material costs	−0.75	Global, with varying intensity in different regions	Medium term (2–4 years)
Religious and cultural restrictions	−0.50	Middle East, North Africa and some Asian countries	Long term (≥4 years)

Source: Authors’ adaptation based on Mordor Intelligence market analyses.

**Table 3:** Comparison of the main technologies used for producing alcohol-free and low-alcohol beer.

Method	Process	Initial ABV (%)	Process Conditions (T, P)	Steps	Final ABV (%)	Aroma and Quality Indicators	Dominant Energy Vector and Cost Factors	Refs.
**Physical**	Vacuum Distillation	4.8–5.5 ^1^	34–50 °C; 60–200 mbar	1	0.05–0.50	Partial losses of volatile esters and higher alcohols; aroma recovery may be integrated.	Thermal energy. Operation at reduced pressure lowers the ethanol boiling point, permitting dealcoholisation at 34–50 °C and reducing thermal input relative to atmospheric-pressure distillation.	[17,37,57,88]
	Spinning Cone Columns (SCC)	≈5.0 ^2^	35–60 °C; pressure nr ^3^	2	0.04–0.50	Aroma recovered in a first stripping stage and reincorporated after ethanol removal; partial reduction in volatile esters and higher alcohols.	Thermal energy. Continuous operation without external reflux; steam demand depends on the number of stripping stages.	[33,86]
	Reverse Osmosis (RO)	4.8–5.5	5–20 °C; 10–40 bar	2	0.45–0.50	Preserves most aroma compounds and mouthfeel; slight loss of bitterness and volatile aroma compounds; post-treatment (e.g., salt/glycerol addition) may be required.	Electrical (pumping) energy to sustain a transmembrane pressure of 10–40 bar; no thermal input. Scalable.	[66,79,87]
	Nanofiltration (NF)	4.7–5.0	19 ± 1 °C; 5–8.6 bar	1	<0.5	Preserves flavour profile and foam stability; loss of monovalent ions and some volatile esters/aldehydes may require post-treatment.	Electrical (pumping) energy at 5–8.6 bar; no thermal input. Mild operating conditions. High initial capital investment.	[60,66]
	Osmotic Distillation (OD)	≈5.0 ^2^	10–20 °C; atmospheric pressure	1	0.40–1.10	Generally associated with higher aroma retention than thermal methods, although partial losses of esters and higher alcohols still occur. Limited changes in colour and bitterness.	Isothermal; negligible thermal input at atmospheric pressure.	[33,71]
**Biological**	Arrested/Limited Fermentation	nr ^3^	Fermentation at 8 °C; atmospheric pressure	1	0.3–1.0	Higher residual sugars and wort-like flavour; reduced formation of esters and higher alcohols; lower fermentation-derived aroma complexity.	No post-fermentation dealcoholisation equipment required. Cooling process required for fermentation control.	[33,49]
	Special Yeast Strains	nr ^3^	Fermentation at 10–20 °C; atmospheric pressure	1	0.05–2.32	Reduced ethanol production through limited maltose/maltotriose utilisation; aroma profile strongly strain-dependent, with several strains producing improved fruity ester profiles compared with arrested fermentation.	No post-fermentation dealcoholisation equipment required. Standard temperature control.	[33,43]

Physical dealcoholisation methods: initial and final ABV % are reported as values or ranges available in the cited literature. Superscript flags denote the origin of each value: ^1^: value calculated by the authors from ethanol concentrations (g L^−1^) reported in the original studies using the following equation: ABV (% *v*/*v*) = ethanol concentration (g L^−1^)/(0.78924 × 10); ^2^: value estimated by the authors when the initial ABV was not explicitly reported. In these cases, an approximate value (≈5% ABV) was adopted to represent the typical alcohol content of conventional beers processed in the cited studies. Unless otherwise indicated by superscripts ^1^ or ^2^, all values reported in the table were taken directly from the cited studies. Biological methods: for fermentation temperature and final ABV % representative values or ranges are reported when multiple studies were summarised. ^3^ nr: not reported.

## Data Availability

No new data were created or analyzed in this study. Data sharing is not applicable to this article.
